# Knockdown resistance in *Anopheles vagus, An. sinensis, An. paraliae *and *An. peditaeniatus *populations of the Mekong region

**DOI:** 10.1186/1756-3305-3-59

**Published:** 2010-07-21

**Authors:** Katrijn Verhaeghen, Wim Van Bortel, Ho Dinh Trung, Tho Sochantha, Kalouna Keokenchanh, Marc Coosemans

**Affiliations:** 1 Department of Parasitology, Institute of Tropical Medicine Antwerp, Nationalestraat 155, B-2000 Antwerpen, Belgium; 2 Department of Entomology, National Institute of Malariology, Parasitology and Entomology, Luong The Vinh street, B.C. 10.200 Tu Liem, Hanoi, Vietnam; 3 Department of Entomology, National Center for Malaria Control, Parasitology and Entomology, 372 Monivong Boulevard, Phnom Penh, Cambodia; 4 Department of Entomology, Center of Malariology, Parasitology and Entomology, Kualuang Road, Vientiane, Laos; 5 Department of Biomedical Sciences, Faculty of Pharmaceutical, Veterinary and Biomedical Sciences, University of Antwerp, Universiteitsplein 1, B-2610 Antwerpen (Wilrijk), Belgium

## Abstract

**Background:**

In the Mekong region (Vietnam, Cambodia and Laos), a large investigation was conducted to assess the susceptibility of Anopheles species against DDT and pyrethroids. In this study, the resistance status of the potential malaria vectors *An. vagus, An. sinensis, An. paraliae *and *An. peditaeniatus *was assessed.

**Methods:**

Bioassays were performed on field collected unfed female mosquitoes using the standard WHO susceptibility tests. In addition, the DIIS6 region of the *para*-type sodium channel gene was amplified and sequenced and four allele-specific PCR assays were developed to assess the *kdr *frequencies.

**Results:**

In Southern Vietnam all species were DDT and pyrethroid resistant, which might suggest the presence of a *kdr *resistance mechanism. Sequence-analysis of the DIIS6 region of the *para*-type sodium channel gene revealed the presence of a L1014S *kdr *mutation in *An. vagus, An. sinensis *and *An. paraliae*. In *An. peditaeniatus*, a low frequency L1014S *kdr *mutation was found in combination with a high frequency L1014F *kdr *mutation. For pyrethroids and DDT, no genotypic differentiation was found between survivors and non-survivors for any of these species. In the two widespread species, *An. vagus *and *An. sinensis*, *kdr *was found only in southern Vietnam and in Cambodia near the Vietnamese border.

**Conclusions:**

Different levels of resistance were measured in Laos, Cambodia and Vietnam. The *kdr *mutation in different *Anopheles *species seems to occur in the same geographical area. These species breed in open agricultural lands where malaria endemicity is low or absent and vector control programs less intensive. It is therefore likely that the selection pressure occurred on the larval stages by insecticides used for agricultural purposes.

## Background

Insecticide resistance may jeopardize the enormous malaria control efforts which have resulted in a significant decrease in the malaria burden in the Mekong region [1]. Insects may survive the toxic effect of insecticides by different resistance mechanisms. The major mechanisms involve either mutations within the target site of the insecticide or an alteration in the rate of insecticide detoxification. The *para*-type sodium channel is the target for both pyrethroids and DDT and mutations in this gene have been linked to knockdown resistance (*kdr*) in several insects [2]. In the malaria vector *Anopheles gambiae sensu lato*, two different mutations at codon 1014 of domain II of the sodium channel gene have been associated with knockdown resistance. The first mutation involves a point mutation resulting in a leucine-to-phenylalanine (L1014F) substitution, whereas a second mutation results in a leucine-to-serine (L1014S) substitution [3,4]. Recently, a leucine-to-cysteine (L1014C) substitution was found in permethrin resistant *An. sinensis *populations of Korea [5]. In the detoxification of insecticides, different enzyme families are involved and elevated levels of esterases, monooxygenases and glutathione-S-transferases (GST) have been linked with insecticide resistance in *Anopheles *[6-9].

In the Mekong countries, a large investigation has been conducted to assess the susceptibility of different *Anopheles *species against pyrethroid insecticides and DDT. Amongst the main malaria vectors, *Anopheles epiroticus *was highly pyrethroid resistant in the Mekong delta, whereas *An. minimus s.l*. was pyrethroid resistant in some localities in northern Vietnam [10]. A low level of phenotypic pyrethroid resistance was found in *An. dirus sensu stricto *from central Vietnam [10]. In these main malaria vectors no *kdr *mutation was observed [11].

Here, the resistance status of the potential vectors *An. vagus, An. sinensis, An. paraliae *and *An. peditaeniatus *was assessed. These species are abundant in the Mekong region and despite their zoophilic trend they regularly bite humans [12] and can play a role in the maintenance of malaria at low or epidemic level [13]. *An. sinensis *contributes to low malaria endemicity in the plains of China [14] and epidemics in North Korea [15]. Based on positive CSP ELISA tests on head and thorax, *An vagus *has been suspected as malaria vector in Bangladesh [16,17], Sri Lanka [13] and in the Assam state of India [18]. Similarly *An. peditaeniatus *was found ELISA positive in Thailand [19] and Sri Lanka [13]. *An. paraliae *and *An. sinensis *are genetically closely related and can be easily confused on morphological characters. *An. sinensis *and *An peditaeniatus *are also vectors of *Brugia malayi *[20,21]. All these species breed in open agricultural lands, like rice fields [22-24] and can be considered as indicator species for insecticide pressure from agricultural origins. In this study, the role of *kdr *mutations in the different resistant *An. vagus, An. sinensis, An. paraliae *and *An. peditaeniatus *populations of the Mekong region was assessed.

## Materials and methods

### Mosquito collections and bioassays

Bioassays were done in the framework of a cross country survey on insecticide resistance in the Mekong region [10]. The list of the study sites with coordinates is giving in Additional file 1. Briefly, from 2003 until 2005 adult female mosquitoes were collected by different collection methods throughout Vietnam, Cambodia and Laos and identified morphologically in the field by use of a standardized key for medically important anophelines [25]. Bioassays were performed using the standard WHO susceptibility test kit with diagnostic concentrations of 0.75% permethrin and 4% DDT [26]. The bioassays were done on adult collected unfed female mosquitoes meaning that the age of the tested specimens was unknown. Additional bioassays were performed with diagnostic concentrations of type II pyrethroids (0.05% lambda-cyhalothrin, 0.082% (30 mg/m^2^) alpha-cypermethrin or 0.05% deltamethrin) and 0.5% etofenprox. Impregnated and control papers were supplied by the Vector Control Research Unit, Universiti Sains Malaysia. The exposure time was 60 min with tubes maintained in the vertical position. After exposure, mosquitoes were kept under observation for 24 h and supplied with 10% sugar solution. Mortality was read after this 24 h period and corrected by Abbott's formula, if the control mortality was between 5 and 20% [26]. The bioassay results were divided into three mortality categories according to the WHO criteria [26]. A 24 h post-exposure mortality less than 80% indicates resistance, whereas a mortality higher than 98% indicates susceptibility. Intermediate mortality levels suggest the possibility of resistance that needs to be confirmed. After the bioassays, the mosquitoes were dried over silica gel.

### Species identification

One to six legs of individual mosquitoes were used for genomic DNA extraction, applying the procedure described in Collins *et al. *[27]. DNA was resuspended in 25 μl TE buffer (10 mM Tris-HCl pH 8; 1 mM EDTA). A negative control was included with every set of extractions. The identification of *An. vagus, An. sinensis, An. paraliae *and *An. peditaeniatus *specimens of which the DIIS6 region of the *para*-type sodium channel gene was sequenced, was confirmed by sequencing of the ITS2 rDNA gene using the primers described in Van Bortel *et al. *[28].

### Detection of *kdr *mutations

**Figure 1:**
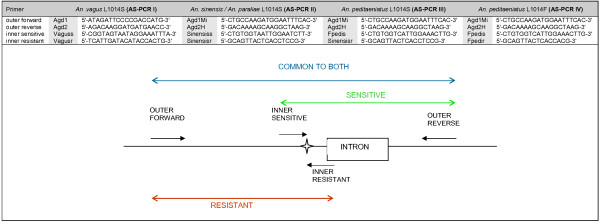
**Schematic representation and the primer sequences of the AS-PCRs used to detect the L1014S *kdr *mutation in *An. vagus *(AS-PCR I), *An. sinensis *(AS-PCR II), *An. paraliae *(AS-PCR II), *An. peditaeniatus *(AS-PCRIII) and the L1014F *kdr *mutation in *An. peditaeniatus *(AS-PCR IV)**. The figure represents the DIIS6 region of the sodium channel gene. The intron is represented by a box and codon 1014 is indicated by a star graphic.

Primers Agd1 and Agd2 [3] were used to amplify the DIIS6 region (*kdr*- region or transmembrane segment 6 of the domain II) of the *para*-type sodium channel gene for *An. vagus*, whereas primers Agd1Mi and Agd2H (Figure 1) amplified the DIIS6 region for *An. sinensis*, *An. paraliae *and *An. peditaeniatus*. Amplification was performed in a 50 μl reaction containing 1 μl of template DNA, 1 × Qiagen PCR buffer, 1 mM MgCl_2_, 200 μM of each dNTP, 100 nM of each primer and 1 unit *Taq *DNA polymerase (Taq PCR core kit, Qiagen, Hilden, Germany).

The cycling conditions were as follows: initial denaturation at 94°C for 3 min, 40 cycles of 1 min denaturation at 94°C, 30 s annealing at 47°C and 30 s extension at 72°C followed by a final extension of 10 min at 72°C. Amplification products were checked on a 2% agarose gel, stained with ethidium bromide and visualised on the Syngene Ingenius LHR (Westburg, Leusden, The Netherlands). The resulting PCR product was cloned by use of the Original TA cloning kit according to the manufacturer's instructions (Invitrogen, Carlsbad, California). Plasmid and direct PCR sequencing were done by the VIB genetic service facility (University of Antwerp, Belgium) and aligned with ClustalW version 1.3 [29].

The DIIS6 sequences were used to develop primers for four allele-specific PCR assays (AS-PCRs) to assess the *kdr *frequencies in the different *Anopheles *populations. An additional A was added to 3'-end of the primer Vaguss in order to minimize self-complementarity and additional mismatch bases were introduced in the Sinensiss and Sinensisr primers at the 4^th ^nucleotide from the 3'-end (A was replaced by T) to obtain more specific results (Figure 1). The AS-PCR assays were optimized by running genomic DNA templates that had been previously genotyped by DNA sequencing. All AS-PCR assays were performed in a 50 μl reaction mixture containing: 1 × Qiagen PCR buffer, 1 × Q solution, 0.5 mM MgCl_2_, 200 μM dNTP's, 400 nM of the outer forward and reverse primer, 500 nM of the inner resistant and sensitive primer (Figure 1), 1 unit Taq DNA polymerase (Qiagen, Hilden, Germany) and 1 μl template. The cycling conditions described above for the amplification of the DIIS6 region were used. The amplification products were electrophoresed on a 3% mixed agarose gel (1.5% agarose and 1.5% small fragment agarose) and visualised under UV light after ethidium bromide staining. In each assay, a sequenced heterozygote resistant mosquito was run with the AS-PCR as positive control.

The *kdr *genotype frequencies of mosquitoes exposed to WHO bioassay were compared for dead and surviving using the exact tests for population differentiation in Genepop (version 3.4) [30].

## Results

### Bioassays

**Figure 2:**
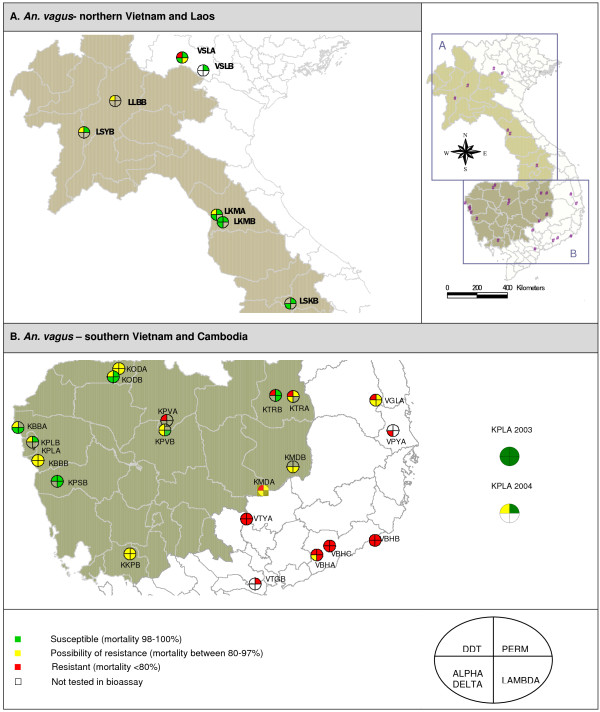
**Mortality categories obtained for the *An. vagus *populations of Vietnam, Laos and Cambodia**. The first quartile (right, up) represents the results obtained with 0.75% permethrin (PERM). In clockwise direction, results with discriminating concentrations of lambda-cyhalothrin (LAMBDA), alpha-cypermethrin (ALPHA) (Vietnam) or deltamethrin (DELTA) (Cambodia and Laos) and DDT are given. The number of exposed mosquitoes varied from 20 (KPSB, lambda-cyhalothrin)* to 296 (LSYB, DDT). * LKMA (perm, delta, alpha); LKMB (perm, delta), KPSB (perm), KPLA (lambda), KTRB (lambda): number of exposed mosquitoes <20.

Details of the bioassays can be found in Additional file 2. DDT resistance was widespread over the *An. vagus *populations of Laos, Cambodia and Vietnam. In Laos and northern Vietnam, the *An. vagus *populations remained pyrethroid susceptible. In Cambodia, the *An. vagus *populations were pyrethroid susceptible or tolerant. In southern Vietnam, the pyrethroid resistance increased and a combination of DDT and pyrethroid resistance was found (Figure 2).

**Figure 3:**
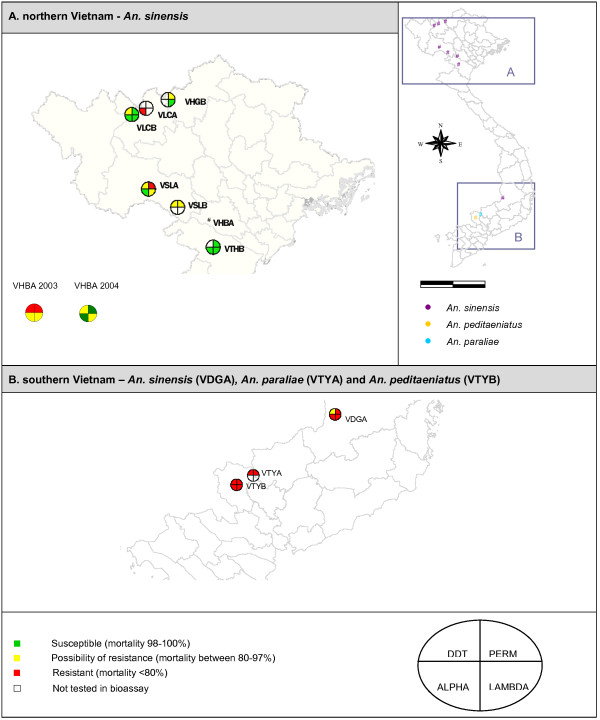
**Mortality categories obtained for *An. sinensis, An. paraliae *and *An. peditaeniatus***. The first quartile (up right) represents the results obtained with 0.75% permethrin (PERM). In clockwise direction, results with discriminating concentrations of lambda-cyhalothrin (LAMBDA), alpha-cypermethrin (ALPHA) and DDT are given. The number of exposed *An. sinensis *varied from 20* (VTYA, permethrin) to 113 (VLCA, alpha-cypermethrin; VTHB, lambda-cyhalothrin) whereas the number of exposed *An. peditaeniatus *varied from 100 to 105. For each insecticide between 20 (VTYA, permethrin) and 73 (VTYA, DDT) *An. paraliae *mosquitoes were exposed. * VHGB (perm): number of exposed mosquitoes <20.

The Vietnamese *An. sinensis *populations were possibly DDT resistant. In southern Vietnam, a combination of DDT and pyrethroid resistance was found for *An. sinensis*, *An. paraliae *and *An. peditaeniatus *(Figure 3).

### *Kdr *mutations

**Figure 4:**
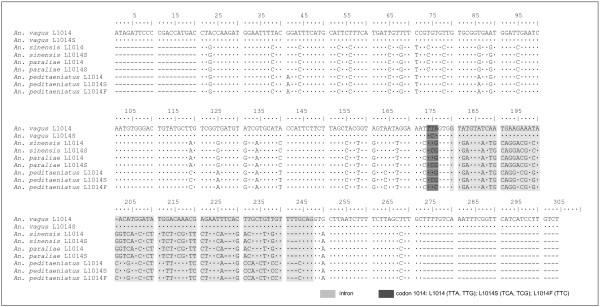
**Alignment of genomic DNA fragments of the DIIS6 region of the *para*-type sodium channel gene obtained for *An. vagus*, *An. sinensis*, *An. paraliae *and *An. peditaeniatus***. The position of the intron was determined by comparison with the mRNA sequences available in GenBank for *An. vagus *and *An. gambiae *[DQ026447; Y13592]. The sequences were aligned with ClustalW version 1.83 [29]. Identical nucleotides are indicated by a point (·), dashes (-) refer to missing nucleotides. At amino acid level the wild type *An. vagus *(L1014), *An. sinensis *(L1014), *An. paraliae *(L1014) and *An. peditaeniatus *(L1014) are 100% identical to the amino acid composition of the DIIS6 region of a wild type *An. gambiae *[Y13592].

Sequences of the DIIS6 region of the *para*-type sodium channel gene were obtained for both, live and dead *An. vagus *(n = 65), *An. sinensis *(n = 33), *An. paraliae *(n = 21) and *An. peditaeniatus *(n = 87) mosquitoes. In pyrethroid and DDT resistant *An. vagus, An. sinensis *and *An. paraliae *populations, a leucine-to-serine replacement at codon 1014 was observed. In *An. peditaeniatus*, both a leucine-to-phenylalanine and a leucine-to-serine replacement were detected at codon 1014. The last amino acid replacement was only detected in combination with the L1014F mutation. To confirm the presence of both *kdr *alleles in *An. peditaeniatus *individuals, cloning and plasmid sequencing was performed (Figure 4).

**Figure 5:**
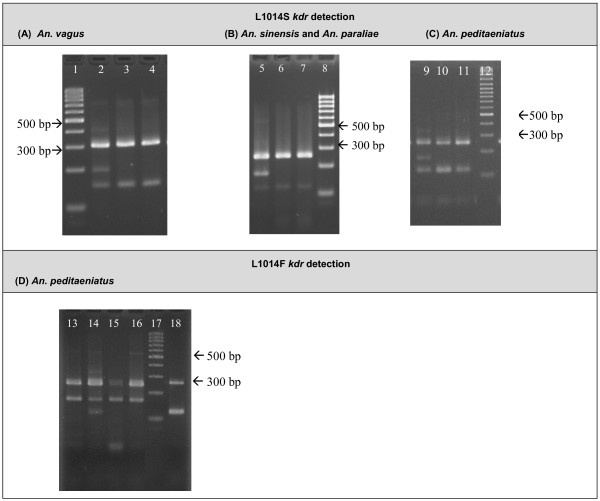
**PCR fragments obtained using the different AS-PCRs after separation on a 3% mixed agarose gel**. **(A)**. Detection of the L1014S mutation in *An. vagus *(AS-PCR I). Lane 1:100 bp ladder; Lane 2: heterozygous specimens (L1014/L1014S); Lane 3 and 4: homozygous wild type mosquitoes (L1014/L1014). **(B) **Detection of the L1014S mutation in *An. sinensis *and *An. paraliae *(AS-PCR II). Lane 5: heterozygous specimens (L1014/L1014S); Lane 6 and 7: homozygous wild type mosquitoes (L1014/L1014); Lane 8: 100 bp ladder. **(C) **Detection of the L1014S mutation in *An. peditaeniatus *(AS-PCR III). Lane 9: heterozygous specimens (L1014/L1014S); Lane 10 and 11: homozygous wild type mosquitoes (L1014/L1014); Lane 8: 100 bp ladder. **(D) **Detection of the L1014F mutation in *An. peditaeniatus *(AS-PCR IV). Lane 13, 15 and 16: homozygous resistant specimen (L1014F/L1014F); Lane 14: heterozygous mosquito (L1014/L1014F); Lane 17: 100 bp ladder; Lane 18: homozygous wild type mosquito (L1014/L1014).

In order to determine the *kdr *frequencies in the Southeast Asian *Anopheles *populations, four different PCR assays were developed to detect the L1014S in *An. vagus *(AS-PCR I), *An. sinensis *and *An. paraliae *(AS-PCR II), *An. peditaeniatus *(AS-PCR III) and the L1014F *kdr *mutation in *An. peditaeniatus *(AS-PCR IV). For the detection of the L1014S mutation, an internal control band (303 bp for *An. vagus*; 255 bp for *An. sinensis*, *An. paraliae *and *An. peditaeniatus*), a specific band for the L1014S *kdr *allele (194 bp in *An. vagus*, 171 bp in *An. sinensis, An. paraliae *and *An. peditaeniatus*) and a band for the wild type L1014 allele were obtained (147 bp for *An. vagus*, 119 bp for *An. sinensis, An. paraliae *and *An. peditaeniatus*) (Figure 5). The AS-PCR IV assay was designed to detect the L1014F *kdr *mutation in *An. peditaeniatus *with a control band of 255 bp, a 171 bp band for the L1014F allele and a 119 bp band for the wild type L1014 allele. The Fpedis primer (sensitive specific) of the AS-PRC IV assay can also anneal to the L1014S allele, hence the L1014S *kdr *allele can be misrecognized as the wild type allele L1014. This non-specific annealing can occur because there is only one nucleotide mismatch between the second nucleotide from the 3'-end of primer Fpedis and the sequence of the L1014S allele (T-C mismatch). For this reason the results of both PCRs (AS-PCR III and IV) needed to be combined in order to genotype the *An. peditaeniatus *specimens correctly.

By use of these AS-PCRs, 3305 *An. vagus *mosquitoes of 28 populations throughout Vietnam, Laos and Cambodia were analysed for the presence of the L1014S *kdr *mutation. The L1014S allele was found only in 7 populations in heterozygote form (L1014S/L1014 genotype) in southern Vietnam and in Cambodia near the Vietnamese border with a frequency varying from 0.7% to 15.7% (Table 1). The highest *kdr *frequency was reported in the VTGB population, which is the most southerly located *An. vagus *population. However, in VTGB the L1014S *kdr *frequency is likely to be overestimated, because only specimens that survived the permethrin WHO bioassay were analysed, whereas in the other *An. vagus *populations, both bioassay survivors and non-survivors were tested.

**Table 1:** Genotype and allele frequencies of the *kdr *alleles found in *kdr *resistant *An. vagus, An. sinensis, An. paraliae *and *An. peditaeniatus *populations

			Genotype frequency (%)						Allele frequency (%)		
					
Anopheles Species	Study site	Number	L1014/L1014	L1014/L1014S	L1014S/L1014S	L1014S/L1014F	L1014/L1014F	L1014F/L1014F	L1014S	L1014F	HWE p-value
*vagus*	KKPB	214	98.13	1.87	0	nd	nd	nd	0.93	nd	1
	KMDA	159	96.86	3.14	0	nd	nd	nd	1.57	nd	1
	VBHC	469	93.60	6.40	0	nd	nd	nd	3.20	nd	1
	VGLA	350	98.57	1.43	0	nd	nd	nd	0.71	nd	1
	VPYA	91	97.80	2.20	0	nd	nd	nd	1.10	nd	1
	VTGB*	35	68.57	31.43	0	nd	nd	nd	15.71	nd	0.5656
	VTYA	405	95.31	4.69	0	nd	nd	nd	2.35	nd	1
											
*sinensis*	VDGA	323	95.36	4.64	0	nd	nd	nd	2.32	nd	1
											
*paraliae*	VTYA	61	47.55	39.34	13.11	nd	nd	nd	32.79	nd	0.3935
											
*peditaeniatus*	VTYB	446	0.67	nd	nd	0.67	2.69	95.97	0.30	97.60	0.0006

All bioassay survivors and non-survivors were analysed. Only populations where *kdr *mutations were found are included in the table. Deviation from Hardy-Weinberg expectation (HWE - exact test using GENEPOP [30]). * Frequency based only on bioassay survivors. nd = not detected.

**Figure 6:**
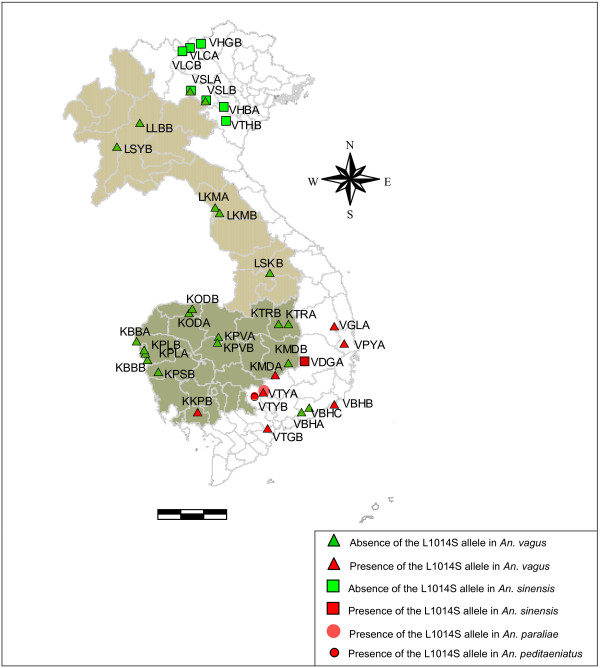
**Geographical distribution of the L1014S *kdr *allele in *An. vagus, An. sinensis*, *An. paraliae *and *An. peditaeniatus***. For each population, at least 20 specimens were analyzed.

By use of the AS-PCR II, 1058 *An. sinensis *specimens of 8 populations were genotyped for the L1014S mutation. The L1014S *kdr *mutation was found only in one population of southern Vietnam at a low frequency (2.3%) (Table 1). In the two widespread *Anopheles *species, *An. vagus *and *An. sinensis*, the geographical distribution of the L1014S *kdr *mutation overlap in southern Vietnam (Figure 6). In *An. paraliae*, the L1014S *kdr *mutation was found at a moderate level (32.8%) in southern Vietnam (VTYA) (Table 1). In *An. peditaeniatus *of southern Vietnam (VTYB), the L1014F *kdr *allele was observed at a high frequency (97.6%, n = 446). The L1014S *kdr *allele was only observed in combination with the L1014F *kdr *allele at a low frequency (0.3%) The three specimens with the L1014S/L1014F genotype survived the discriminative dosage of DDT or lambda-cyhalothrin in the bioassay (Table 2).

**Table 2:** Genotype frequencies found for *An. vagus, An. sinensis, An. paraliae *and *An. peditaeniatus*

Species	Insecticide	Alive	N	*kdr *genotype				**p-value **^**3**^
					
		**Dead **^**2**^		L1014/L1014	L1014/L1014S	L1014S/L1014S		
*An. vagus *^*1*^	DDT	A	160	93.75%	6.25%	0.00%		0.2297
		D	298	96.64%	3.36%	0.00%		
	PERMETHRIN	A	105	87.86%	12.14%	0.00%		0.3844
		D	334	96.71%	3.29%	0.00%		
	ALPHA-CYPERMETHRIN	A	134	94.78%	5.22%	0.00%		0.0970
		D	205	98.54%	1.46%	0.00%		
	LAMBDA-CYHALOTHRIN	A	104	95.19%	4.81%	0.00%		0.7211
		D	103	97.09%	2.91%	0.00%		

*An. sinensis*	DDT	A	16	93.75%	6.25%	0.00%		1.0000
		D	46	93.48%	6.52%	0.00%		
	PERMETHRIN	A	44	90.91%	9.09%	0.00%		0.3642
		D	39	97.44%	2.56%	0.00%		
	ALPHA-CYPERMETHRIN	A	40	97.50%	2.50%	0.00%		1.0000
		D	43	95.35%	4.65%	0.00%		
	LAMBDA-CYHALOTHRIN	A	45	95.56%	4.44%	0.00%		1.0000
		D	31	96.77%	3.23%	0.00%		

*An. paraliae*	DDT	A	21	38.09%	42.86%	19.05%		0.6918
		D	29	44.83%	41.38%	13.79%		
	PERMETHRIN	A	30	50.00%	50.00%	0.00%		1.0000
		D	3	66.67%	33.33%	0.00%		

				L1014/L1014	L1014/L1014F	L1014F/L1014F	L1014F/L1014S	
					
*An. peditaeniatus*	DDT	A	93	1.08%	6.45%	90.32%	2.15%	0.7497
		D	7	0.00%	0.00%	100.00%	0.00%	
	PERMETHRIN	A	74	1.35%	0.00%	98.65%	0.00%	1.0000
		D	12	0.00%	0.00%	100.00%	0.00%	
	ALPHA-CYPERMETHRIN	A	77	0.00%	2.60%	97.40%	0.00%	0.3085
		D	10	0.00%	10.00%	90.00%	0.00%	
	LAMBDA-CYHALOTHRIN	A	79	0.00%	2.53%	96.20%	1.27%	1.0000
		D	10	0.00%	0.00%	100.00%	0.00%	
	ETOFENPROX	A	73	1.37%	1.37%	97.26%	0.00%	1.0000
		D	8	0.00%	0.00%	100.00%	0.00%	

For *An. vagus*, the genotypes of the different populations could be pooled, because for each population the *kdr *genotypes were equally distributed among the bioassay survivors and non-survivors (exact test for population differentiation using GENEPOP [30]). ^1 ^Pooled results for *An. vagus *populations of KMDA, KKPB, VBHC, VGLA, VPYA and VTYA. The An. vagus population of VTGB was excluded from the analysis, because only permethrin survivors were available. ^2 ^A: Alive, D: Dead; 24 hours after exposure to a discriminative dosage of an insecticide in a WHO bioassay. ^3 ^p-value of the genotypic differentiation between survivors and non-survivors (* correlation is significant at the 0.05 level) (Genepop; [30]).

To assess the role of the *kdr *mutations in conferring resistance, a comparison was made between the different *kdr *genotypes and the survival status in the bioassay. For the four species, the different *kdr *genotypes were equally distributed among bioassay survivors and non-survivors, even when homozygote resistant specimens were present (Table 2).

## Discussion

From 2003 to 2005, WHO bioassays were performed on the *An. vagus, An. sinensis, An. paraliae *and *An. peditaeniatus *populations of Vietnam, Laos and Cambodia to assess their susceptibility to DDT and pyrethroids. Different levels of pyrethroid and DDT resistance were found. In the present bioassays, field collected females of unknown age were used, whereas the WHO protocol recommends the use of 1-3 day old females. As mortality in bioassays is significantly higher with old females compared with young ones [31] the results of the current study only underestimate the resistance problem.

In southern Vietnam, *An. vagus, An. sinensis*, *An. peditaeniatus *and *An. paraliae *were highly resistant to both DDT and pyrethroids, which might suggest the presence of *kdr*. Sequences of the DIIS6 region of the *para*-type sodium channel gene, revealed the presence of a L1014S *kdr *mutation in *An. vagus, An. sinensis, An. paraliae *and *An. peditaeniatus *and a L1014F *kdr *mutation in *An. peditaeniatus*. In the two widespread species, *An. vagus *and *An. sinensi*s, knockdown resistance was observed only in Cambodia near the Vietnamese border and in southern Vietnam. In these species, the L1014S *kdr *allele was found at a low frequency and only in the heterozygous form. In *An. paraliae*, the L1014S *kdr *frequency was higher and both heterozygous and homozygous resistant mosquitoes were observed. In *An. peditaeniatus*, the L1014S *kdr *allele was very rare (0.3%) and was found only in combination with the common L1014F *kdr *allele (97.6%). Interestingly, in *An. gambiae s.s*. of Uganda, Equatorial Guinea, Gabon and Cameroon, specimens were found to carry the same *kdr *mutations in a heterozygous state (L1014F/L1014S genotype) [32-35].

For *An. vagus*, *An. sinensis*, *An. paraliae *and *An. peditaeniatus*, the different *kdr *genotypes were equally distributed among bioassay survivors and non-survivors. For *An. vagus *and *An. sinensis *this could be expected since *kdr *is a recessive trait [36,37] and the L1014S *kdr *mutation was only found in the heterozygous form. In *An. paraliae*, however, homozygous resistant L1014S/L1014S mosquitoes were found and equally distributed among bioassay survivors and non-survivors. Also in the *An. peditaeniatus *population where the L1014F *kdr *allele occurs at high allelic frequency, no connection was found between the genotype and resistant phenotype. In *An. peditaeniatus*, even homozygous resistant L1014F/L1014F mosquitoes were found among the dead mosquitoes which suggest that beside the L1014F *kdr *mutation other subsequent mutations in the *para*-type sodium channel gene might be needed for a mosquito to survive an exposure to a discriminating concentration of an insecticide. Such secondary mutations were found in *Haematobia irritans *and *Musca domestica *populations where super-*kdr *mutations (M918T) in the DIIS4-DIIS5 linker of the *para*-type sodium channel enhanced the pyrethroid resistance of individuals with the L1014F *kdr *mutation [38,39]. Because in the present work only the DIIS6 region of the *para*-type sodium channel gene was sequenced, the presence of additional mutations in the *para*-type sodium channel gene can not be excluded. However, to date, no additional mutations were described in *Anopheles *species.

In the widespread *An. vagus *and *An. sinensis, kdr *was geographically limited to an area in Southern Vietnam. In northern Vietnam, other resistance mechanisms could be involved. A limited number of populations were screened by biochemical assays (results not shown). Preliminary biochemical assays on DDT resistant *An. vagus *and *An. sinensis *populations revealed a high GST activity in Northern and Southern Vietnam. High esterase activity was found in pyrethroid resistant *An. vagus *(VBHA and VBHB) and *An. sinensis *(VHBA 2003, VLCA, VSLA, VSLB) populations without *kdr*, whereas elevated levels of esterase activity were not detected in a kdr resistant population (*An. vagus*: VTYA) (data not shown). This shows that beside knockdown resistance, other mechanisms of insecticide resistance should be systematically explored.

Resistance to insecticides in malaria vectors has been often related to the use of insecticides in agronomy [40] and seems most likely to have developed as a consequence of selection pressure on larvae [41]. The fact that in the same geographical area (southern Vietnam and Cambodia near the Vietnamese border) the *kdr *resistance mechanism was selected in different *Anopheles *species (*An. vagus*, *An. sinensis*, *An. paraliae *and *An. peditaeniatus*) with a similar breeding ecology points in the same direction. *An. vagus*, *An. sinensis*, *An. paraliae *and *An. peditaeniatu*s breed in rice fields [22,23,42] which can be exposed to agricultural insecticides. In this study, investigators failed to collect correct information on pesticide use at household or communal level. However, in Vietnam, the pesticide use in rice fields accounted for 65.5% of the total market value of pesticides in 1996. The pesticide use was the highest in Southern Vietnam where there is a great tendency towards the application of cheaper, hazardous pesticides, including DDT [43,44]. The use of these insecticides in agriculture can explain why DDT resistance still exists in these vectors.

The complex insecticide resistance pattern varying with species and region demonstrates that insecticide resistance in *Anopheles *species of the Mekong region is a complex and dynamic process. Knowledge on the factors which determine insecticide resistance will be necessary to guide an efficient use of insecticides in both public health and agriculture.

## Competing interests

The authors declare that they have no competing interests.

## Authors' contributions

MC and WVB designed the study; revised and supervised the work at all stages. KV carried out the molecular work and drafted the manuscript. HDT, TS and KK supervised the bioassays in the field. All authors read and approved the final manuscript.

## Supplementary Material

Additional file 1 **Coordinates in Decimal Degree of the different collecting sites in Vietnam (V), Cambodia (K) and Laos (L)**. Click here for file

Additional file 2 **Details of the bioassays using the discriminating concentrations of insecticides: 4% DDT, 0.75% permethrin, 0.05% lambda-cyhalothrin, 0.082% (30 mg/m²) alpha-cypermethrin, 0.05% deltamethrin, 05% etofenprox**. Mortalities (after 24 h) and proportion Knocked Down after 60 min exposure (in %). Click here for file
